# Reducing inequities in maternal and child health in rural Guatemala through the CBIO+ Approach of Curamericas: 5. Mortality assessment

**DOI:** 10.1186/s12939-022-01757-7

**Published:** 2023-02-28

**Authors:** Henry B. Perry, Ira Stollak, Ramiro Llanque, Annah Okari, Carey C. Westgate, Alexis Shindhelm, Victoria B. Chou, Mario Valdez

**Affiliations:** 1 Health Systems Program, Department of International Health, Johns Hopkins Bloomberg School of Public Health, Baltimore, Maryland USA; 2 Curamericas Global, Raleigh, North Carolina USA; 3 Consejo de Salud Rural Andino/Curamericas, La Paz, Bolivia; 4 Traveling Nurse, Raleigh, North Carolina USA; 5 Community Health Impact Coalition, New York, New York USA; 6 Department of Neurology, Duke University Medical Center, Durham, North Carolina USA; 7 Global Disease Epidemiology and Control Program, Department of International Health, Johns Hopkins Bloomberg School of Public Health, Baltimore, Maryland USA; 8 Curamericas/Guatemala, Calhuitz, Huehuetenango, San Sebastián Coatán Guatemala

**Keywords:** Maternal mortality, Child mortality, Mortality assessment, Maternal health, Child health, Community health, Primary health care, Community-based primary health care, Implementation research, Census-Based, Impact-Oriented Approach, Care Groups, Community birthing centers, Guatemala, Equity, Curamericas Global, Curamericas/Guatemala

## Abstract

**Background:**

The Curamericas/Guatemala Maternal and Child Health Project, 2011–2015, implemented the Census-Based, Impact-Oriented Approach, the Care Group Approach, and the Community Birthing Center Approach. Together, this expanded set of approaches is known as CBIO+. This is the fifth of 10 papers in our supplement describing the Project and the effectiveness of the CBIO+ Approach. This paper assesses causes, levels, and risk factors for mortality along with changes in mortality.

**Methods:**

The Project maintained Vital Events Registers and conducted verbal autopsies for all deaths of women of reproductive age and under-5 children. Mortality rates and causes of death were derived from these data. To increase the robustness of our findings, we also indirectly estimated mortality decline using the Lives Saved Tool (LiST).

**Findings:**

The leading causes of maternal and under-5 mortality were postpartum hemorrhage and pneumonia, respectively. Home births were associated with an eight-fold increased risk of both maternal (*p* = 0.01) and neonatal (*p* = 0.00) mortality. The analysis of vital events data indicated that maternal mortality declined from 632 deaths per 100,000 live births in Years 1 and 2 to 257 deaths per 100,000 live birth in Years 3 and 4, a decline of 59.1%. The vital events data revealed no observable decline in neonatal or under-5 mortality. However, the 12–59-month mortality rate declined from 9 deaths per 1000 live births in the first three years of the Project to 2 deaths per 1000 live births in the final year. The LiST model estimated a net decline of 12, 5, and 22% for maternal, neonatal and under-5 mortality, respectively.

**Conclusion:**

The baseline maternal mortality ratio is one of the highest in the Western hemisphere. There is strong evidence of a decline in maternal mortality in the Project Area. The evidence of a decline in neonatal and under-5 mortality is less robust. Childhood pneumonia and neonatal conditions were the leading causes of under-5 mortality. Expanding access to evidence-based community-based interventions for (1) prevention of postpartum hemorrhage, (2) home-based neonatal care, and (3) management of childhood pneumonia could help further reduce mortality in the Project Area and in similar areas of Guatemala and beyond.

## Background

This is the fifth paper of our 10-paper series on the Curamericas/Global application of the Expanded Census-Based, Impact-Oriented (CBIO+) Approach to improving the health and well-being of mothers and children in the western highlands of Guatemala. Here, we present a mortality assessment of the Curamericas/Guatemala Maternal and Child Health Project (hereafter referred to as the Project) that was carried out between 2011 and 2015, including a review of the data regarding changes in under-5 and maternal mortality during the period of Project implementation. This paper specifically addresses the following research hypothesis identified in Paper 2 of this series [1]: The CBIO+ Approach reduces mortality of children younger than 5 years of age and maternal mortality relative to (a) baseline measures of these indicators, (b) measures in a comparison area, (c) measures in selected nearby municipalities where the Project was not working, and (d) the overall rural population of the Department of Huehuetenango.

Previous articles in this series described the CBIO+ Approach, the Project, and its setting [2]; study design and methods; changes in the coverage of key maternal and child health indicators [3]; and changes in the nutritional status of children [4]. Subsequent articles discuss the quality of maternity care provided at Community Birthing Centers called *Casas Maternas Rurales* [5], the effects of CBIO+ on women’s empowerment [6, 7], an assessment of the CBIO+ Approach by internal stakeholders [8], and a final concluding article with a cost analysis and a summary of the findings and their broader implications [9]. We turn in this paper to mortality assessment.

Briefly, the non-governmental organization (NGO) Curamericas/Guatemala implemented a set of community-based interventions with community collaboration in an isolated rural mountainous region of northwestern Guatemala in the Department of Huehuetenango with an indigenous Mayan population of 98,000 people. The CBIO approach involves, among other things, conducting a participatory community census and visiting all homes on a regular basis, registering vital events, addressing the epidemiological priorities, and monitoring changes in health status over time [2, 10]. The term CBIO+ refers to the addition of two additional components to the CBIO approach: Care Groups and Community Birthing Centers, also described further in Paper 1 [2]. Care Groups consist of 5–15 Care Group Volunteers (called *Comunicadoras/*Communicators) who are each responsible for 10–15 households. Care Group Promoters (called *Facilitadoras Comunitarias/*Community Facilitators) share health education messages every two weeks with the Care Groups Volunteers who then convey them to the women in their catchment area. The Community Birthing Centers provide an alternative to home delivery, which was almost universal at the outset of the Project. These Birthing Centers are staffed by trained healthcare providers and welcomed the participation of *comadronas* (traditional birth attendants), who are still a vital part of the Maya culture.

The traditional CBIO Approach (from which CBIO+ derives its name) involves several interrelated components, including engaging the community in a mortality assessment based on local surveillance data acquired through routine systematic visitation of all homes. As defined in the CBIO description [10], this mortality assessment helps determine local epidemiological priorities and tailors the program implementation plan to address the leading causes of readily preventable or treatable causes of mortality. Over time, it becomes possible to assess whether health has improved by measuring changes in mortality rates and causes and determining whether any decline can be plausibly attributed to Project activities. By combining Care Groups with CBIO and working with Care Group Volunteers and other community stakeholders (Community Facilitators, *comadronas*, and Village Health Committees), it was possible to establish Vital Events Registers of births, deaths, and causes of death from which the data for the mortality assessment were obtained.

## Methods

### Region and catchment area

The Project was implemented in the entire municipalities of San Sebastián Coatán, Santa Eulalia, and San Miguel Acatán, which are located in the Department of Huehuetenango in the Cuchumatanes mountains. The Project Area was divided into two approximately equal parts. Project implementation began in Area A in 2011 and in Area B in 2013. As described in Paper 2, Areas A and B were geographically and socio-culturally similar. The Project did not have the resources to begin implementation in both areas in 2011. Area A was prioritized for equity considerations since its communities were generally further from existing health clinics. Further details are provided in Papers 1 and 2 in this series [2]. Because Area B received services for a shorter period of time, we were able to look for a “dose-response” effect, meaning that a smaller impact in Area B compared to Area A helps to build the case that the Project activities were responsible for the effects obtained.

### Vital events registration and verbal autopsy data

The methods for collection of vital events and verbal autopsy data are described in the second paper in this series . Here we provide additional details.

The Project created and maintained Vital Events Registers for registering the live births and maternal and child deaths in the communities served. All deaths of children and women of reproductive age were followed up by a verbal autopsy with the family of the deceased. The verbal autopsy was conducted by a higher-level Project staff member (an Institutional Facilitator, who was an experienced graduate nurse) who determined the cause of death and contributing factors. Causes of maternal and child mortality were then derived from these data and used to determine local epidemiological priorities, appropriate interventions, and the impact of the CBIO+ Approach on mortality reduction.

To assess the effectiveness of the CBIO+ Approach on mortality, we calculated changes in maternal mortality and under-5 mortality in the Project Areas and compared this to mortality changes in non-Project areas. Because the Project was implemented in two phases (as described in Paper 2 of this series [1]), with Area A receiving project services from October 2011 to May 2015 and Area B only from October 2013 to May 2015, we were also able to look for a dose-response effect.

**Fig. 1:**
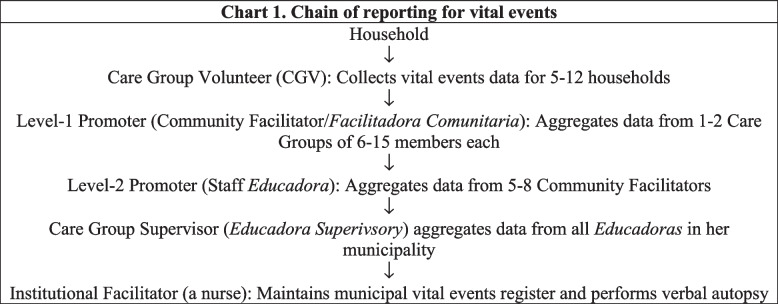
Chain of reporting for vital events

The chain of reporting for vital events data began at the household level. Care Group Volunteers met twice monthly in groups of 5–12 with a Care Group Promoter to learn lessons on health-promoting behaviors and to share data on any new pregnancies, births, maternal deaths, under-5 deaths, and other deaths. The Care Group Promoter then shared these data with the Project *Educadora* assigned to that community, and the Project *Educadora* then passed the data on to her Municipal Care Group Supervisor, who collated the data from all of her *Educadoras* and conveyed the collated data to the municipality’s Institutional Facilitator, who then entered the data into the MS Excel-based Vital Events Register and followed up to perform a verbal autopsy, whose findings were also entered into the Register. It should also be noted that registration of vital events was also a community-wide event, with *comadronas* and village health committee members also reporting vital events as well. This is illustrated in Fig. 1.

Each municipality had two Vital Events Registers maintained as MS Excel files. One Register covered the communities in the municipality that were in Area A, and the other Register covered the communities in the municipality that were in Area B. Registers contained information on (1) all pregnancies and pregnancy outcomes (miscarriage, stillbirth, or live birth); (2) under-5 deaths; (3) deaths among reproductive-age women; and (4) a general mortality registry including deaths of older children, men, and women of non-reproductive age. Each of these events was assigned a unique 12-digit identifier to prevent the duplication of data and track the location of specific vital events in the Register. In the case of a reported maternal or child death, the Institutional Facilitator followed up within two weeks to perform a verbal autopsy with the family of the deceased. Every entry into the Vital Events Register was reviewed for completeness, internal consistency, and accuracy by the Project’s Institutional Facilitator Supervisor. The data were then analyzed each Project Year (PY), which began in October and ended in September.

The verbal autopsy process utilized the standard verbal autopsy form of the Ministry of Health (formally the *Ministerio de Salud Pública y Asistencia Social/*MSPAS) to promote alignment between the Project’s data and MSPAS data [11]. Findings from the verbal autopsy were added to the maternal and under-5 death logs in the Vital Events Register. Types of information gleaned through the verbal autopsy process included classification of the cause of death, location of death, location of delivery, and which of the four “delays” (described further below) may have contributed to the death.

#### Cause of death

The Project used a system of “primary” and “secondary” classifications for cause of death. The primary classification system for maternal deaths utilized the categories used by the MSPAS in their national studies of maternal mortality. These included hemorrhage, pre-eclampsia/eclampsia, sepsis, and other direct and indirect causes. Primary classifications for child death included birth asphyxia, complications of prematurity, pneumonia, diarrhea, sepsis/other infections, and other miscellaneous causes. The secondary classification system was used to classify the primary attributable cause of death further, such as a retained placenta as a cause of hemorrhage, aspiration of meconium for birth asphyxia, or respiratory distress syndrome for complications of prematurity.

#### The four “delays”

For our analysis of factors contributing to maternal deaths, we adopted the MSPAS four-“delay” typology which includes delays in (1) recognizing danger signs, (2) taking action in response to danger signs, (3) reaching a medical facility, and (4) obtaining appropriate medical care once the facility is reached [12]. Though these four delays were developed for maternal deaths, the Project elected to apply them to deaths in under-5 children as well.

Assessing how many deaths are attributable to the first delay helped assess the penetration of the behavior change communications (BCC) that the Project provided through the Care Groups. Quantifying the extensiveness of second delays helped indicate that while the BCC may have been effective in creating awareness of danger signs, there were still other factors that impeded proper care-seeking by the family despite their recognition of the danger. The third delay helped to understand the extent to which transportation was a bottleneck to care, and the fourth delay reflected deficiencies at the referral facility.

### Lives saved tool modeling

The Lives Saved Tool (LiST) is a mathematical modeling tool that estimates the impact of changes in the coverage of evidence-based interventions on mortality in low- and middle-income countries (LMICs) [13]. The LiST Subnational Wizard (Spectrum version 6.00 15 Dec 2020) was used to estimate the impact of the improved coverage of evidence-based interventions on the neonatal mortality rate (NNMR), the under-five mortality rate (U5MR), and the maternal mortality ratio (MMR). The methods and assumptions of the LiST tool have been previously described [14], and the tool has been used for program planning and evaluation [15–18]. The model has also been used to estimate mortality at the subnational level in a LMIC setting [19].

We modeled baseline mortality rates for the Project Area using a combination of data collected by the project and subnational Reproductive Health Survey (RHS) mortality data for the *Noroccidente* (Northwest) Region where project-collected data were unavailable [20]. Municipal census data for the Department of Huehuetenango from 2008 to 2010 were used to establish the baseline population for the projection [21]. Project coverage data at baseline and endline for Project Area A are shown in Table 1 in Paper 3 [3]. See Appendix 5 in Table 14 for a description of the LiST indicators included in our analysis.

**Table 1:** Number of live births, maternal deaths, and maternal mortality ratios (MMRs) in Area A and Area B communities, October 2011–May 2015

Project Year(s)		Communities											
		Area A communities				Area B communities				Area A and B communities combined			
		No. of live births registered	No. of maternal deaths registered	MMR	95% CI	No. of live births registered	No. of maternal deaths registered	MMR	95% CI	No. of live births registered	No. of maternal deaths registered	MMR	95% CI
a. Each year of Project activities													
1	Oct 2011 - Sept 2012	1337	7	524	(128, 919)								
2	Oct 2012 - Sept 2013	1352	10	740	(272, 1207)								
3	Oct 2013 - Sept 2014	1426	4	281	(0, 561)	1149	5	435	(46, 824)	2575	9	350	(117, 583)
4	Oct 2014 - May 2015	906	2	221	(0, 533)	961	6	624	(115, 1134)	1867	8	428	(126, 731)
b. Consolidated years and total													
1–2	Oct 2011 - Sept 2013	2689	17	632	(326, 939)								
3–4	Oct 2013 - Sept 2015	2332	6	257	(47,467)	2110	11	521	(207, 836)	4442	17	383	(197, 568)
1–4	Oct 2011 – May 2015	5021	23	458	(267, 649)	2110	11	521	(207, 836)	7131	34	477	(313, 640)

*Note*: *CI* confidence interval

### Calculation of 95% confidence intervals for mortality data

The vital events registration included all of the births and deaths in the Project Area. Thus, there is, in a sense, no need to calculate confidence intervals since the results represent the values for the entire population rather than for a sample of the population. However, because of the small number of deaths that were registered as a result of the small Project population size and the relative infrequency of deaths, there is another issue that arises, referred to by some as “the instability of small numbers.” Selvin [22] has proposed a method of calculation of 95% confidence intervals for mortality rate estimates that are calculated from a small number of deaths in situations in which no sampling is involved. The method is as follows:

q = probability of death

D = number of deaths

n = population at risk (i.e., number of live births)

q = D/n

$$\hat{\text{q}}=\text{q}\ \text{when}\ \text{q}\ \text{is}\ \text{very}\ \text{small}\ \left[\text{i}.\text{e}.,\left(1-\text{q}\right)\approx 1\right]$$

$$\text{Variance}\ \hat{\text{q}} \approx \frac{\text{q}\left(1-\text{q}\right)}{\text{n}}\approx \frac{\hat{\text{q}}}{\text{n}}=\frac{D}{n\ast n}$$

$$\text{Standard}\ \text{error}\ \text{of}\ \hat{\text{q}}\approx \frac{\sqrt{\text{D}}}{n\ast n}$$

To estimate the 95% confidence intervals for U5MRs and MMRs, the following formula was used:

$$95\%\textrm{CI}=\frac{\textrm{D}\pm 2\sqrt{\textrm{D}}}{\textrm{n}}$$

## Findings

### Assessment of mortality

We present here for maternal deaths, neonatal deaths, and deaths of children < 60 months of age the available data arising from the Register of Vital Events for level of mortality, causes, risk factors, changes over time, and comparisons of mortality between Areas A and B. We also present some data for overall mortality of under-5 children.

### Maternal mortality

#### Level

Between October 2011 and May 2015, the Project registered in Areas A and B 7131 live births and 34 maternal deaths yielding an MMR for the entire Project Area during the time in which vital events were registered of 477 per 100,000 live births with a 95% confidence interval of 313 to 640 per 100,000 live births (Table 1b).

#### Causes

**Fig. 2:**
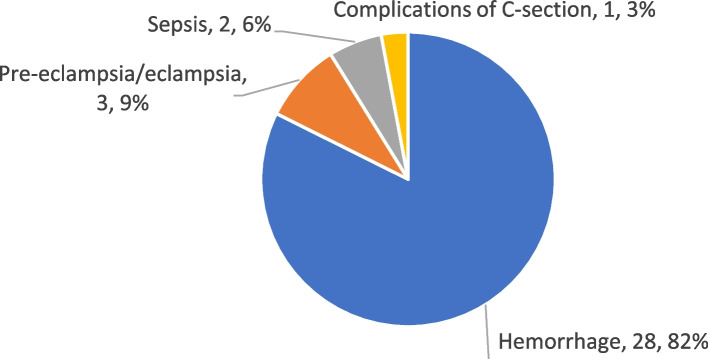
Causes of maternal mortality in Areas A and B combined, October 2011–May 2015. Note: C-section: Cesarean section

Verbal autopsies were obtained for all 34 registered maternal deaths. Hemorrhage, responsible for 82% of deaths, was the leading cause of maternal deaths. Pre-eclampsia/eclampsia was the second leading cause, responsible for 9% of deaths (Fig. 2). No abortion-related maternal deaths were identified.

The most common cause of hemorrhage was retained placenta, which was the underlying factor for 75% (*n* = 21) of all deaths from hemorrhage, followed by uterine atony (18%, *n* = 5) and uterine rupture (7%, n = 2), as shown in Appendix 1 in Table 5.

#### Causes of non-maternal mortality among women of reproductive age

The Project registered the deaths of everyone of all ages. All deaths among women of reproductive age received a verbal autopsy. For deaths that were classified as non-maternal, the causes are shown in Appendix 1 in Table 6. The leading causes of death were cancer, diabetes, pneumonia, and suicide.

#### Risk factors

##### Location of delivery and maternal death

**Fig. 3:**
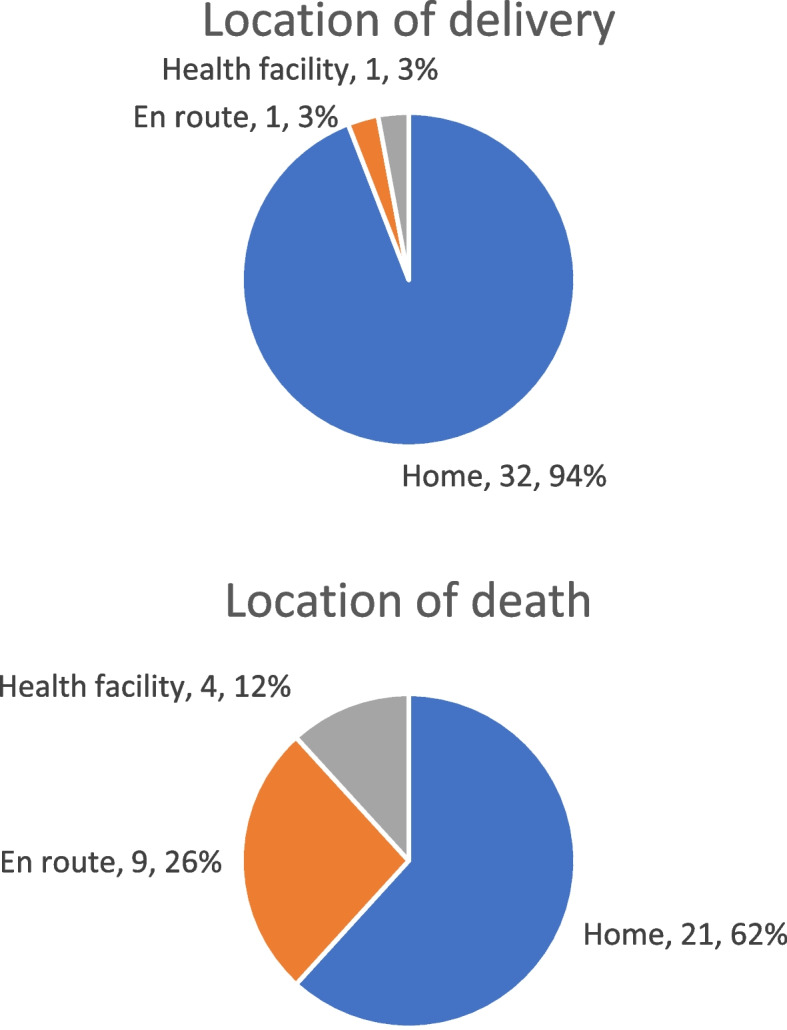
Location of delivery and location of death for women in Areas A and B whose deaths were registered in the Project’s vital events registration system and classified as maternal, October 2011–May 2015

For all maternal deaths, the locations of delivery and death were recorded. These are not necessarily the same since some women who delivered at home sought help following delivery if a complication had developed and then died at a different location. For Areas A and B combined, 94% (*n* = 32) of maternal deaths were among women who delivered at home. For some women who delivered at home and had a complication, care was sought outside of the home. Thus, 26% (*n* = 9) of maternal deaths were among women who died en route to a health facility (following a home delivery), and 12% (*n* = 4) of women died at a health facility (Fig. 3).

Unfortunately, our vital events register did not record the place of birth for all births in the Project Area. However, on the basis of the coverage surveys in Areas A and B and baseline and endline, we estimate that 80% of the births during the period of Project operations took place at home. This estimate of 80% was derived from the following: Using the data from Paper 3 containing measures of coverage of key interventions [3], the overall average of the births attended at home for the baseline and endline KPC surveys in both Areas A and B was 80.0%. Thus, the MMR for home births is estimated to be 578.4 maternal deaths per 100,000 live births (33/5705 * 100,000) compared to an MMR of 70.1 per 100,000 live births for facility-based births (1/1426 * 100,000), yielding an increased risk for a home birth of 8.3 times that of the risk of a facility birth (*p* = 0.01).

##### “Delays” in seeking care

For each maternal death that was registered in our vital events system, one of four delays was assigned as the main contributor to the death by those conducting the verbal autopsy. In nearly all maternal deaths, time is a critical factor, especially in the case of hemorrhage as a woman can bleed to death very quickly during childbirth. Of the four delays, the first delay was assigned as the main contributor in 29% (*n* = 10) of the deaths, with the family or the *comadrona* not recognizing that the woman was in danger. In another 29% (n = 10) of the deaths, the family or the *comadrona* recognized the presence of a danger sign but either chose not to transport the woman to a health facility or experienced a delay in obtaining transportation. The verbal autopsies did not always capture the reason given for the delay in transport, but the most frequently cited reason was lack of money to pay for transportation. Other causes cited were “it is God’s will that she die,” and in one instance, inter-community conflict impeded arranging transportation. The third delay, delay as a result of transport time, was considered to be the main contributor to the death in another 29% (*n* = 10) of deaths. In fact, in nine maternal deaths, the woman died en route to the hospital that was 3-4 hours away over rough mountain roads. The fourth delay, delay in receiving care once at the health facility or inadequate treatment, was considered to be the main contributor to only 12% (*n* = 4) of deaths (see Appendix 2 in Fig. 8).

##### Maternal age

As shown in Appendix 2 in Fig. 9, age at death is fairly evenly distributed, with the youngest being 16 years of age and the oldest 46 years of age. Unfortunately, we do not have data on the ages of all mothers who gave birth and therefore are unable to compute age-specific MMRs.

#### Changes over time

As shown in Table 1a and b, the MMR in Area A declined from 740 deaths per 100,000 live births in Year 2 to 221 deaths per 100,000 live births in Year 4. In Table 1b, we combined data and show the results for the first two years of Project implementation and for the final two years. The decline in the MMR in Area A (632 to 257), a decline of 59.1%, was statistically significant (*p* = 0.005).

We also used LiST to estimate indirectly the number of lives of pregnant women saved in Project Area A on the basis of changes in the coverage of evidence-based maternal health interventions. This analysis, described further in Appendix 5, estimates a maternal mortality decline of 20% compared to an 8% decline that was projected in the absence of the Project, yielding a net decline of 12%.

#### Comparisons between Areas A and B

As shown in Table 1 for Years 3 and 4 combined, the MMR in Area A was half that in Area B (257 per 100,000 live births versus 521 per 100,000 live births). However, this difference is not statistically significant.

#### Comparisons with areas outside of the Project area

The 2014–2015 national Demographic and Health Survey (DHS) reported a national MMR of 140 per 100,000 live births [23]. The MMR in the Project Area was 477 per 100,000 live births, 3.4 times higher. The MMR in Area A during the final two years of the Project (257 per 100,000 live births) was still 1.8 times greater than the national average.

#### The contribution of community birthing centers to reduction in maternal mortality

According to the Community Birthing Center records, only 12.7% of the 7131 deliveries reported in the vital events registry took place at a Birthing Center (Appendix 3 in Table 10)*.* Thus, their contribution to an overall maternal mortality reduction in the Project Area would have been modest at best.

### Neonatal mortality

#### Level

During the life of the Project, the vital events registration system recorded 138 neonatal deaths for 7131 live births, yielding an overall NNMR of 19 per 1000 live births with a 95% confidence interval of 16 to 23 per 1000 live births (Table 2b).

**Table 2:** Number of births, neonatal deaths, and neonatal mortality rates (NNMRs) in Areas A and B communities by Project Year, October 2011–May 2015

Project Year(s)		Communities											
		Area A communities				Area B communities				Area A and B communities combined			
		No. of live births registered	No. of neonatal deaths registered	NNMR	95% CI	No. of live births registered	No. of neonatal deaths registered	NNMR	95% CI	No. of live births registered	No. of neonatal deaths registered	NNMR	95% CI
a. Each year of Project activities													
1	Oct 2011- Sept 2012	1337	22	16	9, 23								
2	Oct 2012 Sept 2013	1352	27	20	12, 28								
3	Oct 2013- Sept 2014	1426	17	12	6, 18	1149	18	16	8, 23	2575	35	14	9, 18
4	Oct 2014- May 2015	906	34	38	25, 50	961	20	21	12, 30	1867	54	29	21, 37
b. Consolidated years and total													
1–2	Oct 2011- Sept 2013	2689	49	18	13, 23								
3–4	Oct 2013- Sept 2015	2332	51	22	16, 28	2110	38	18	12, 24	4402	89	20	16, 25
1–4	Oct 2011- May 2015	5021	100	20	16, 24	2110	38	18	12, 24	7131	138	19	16, 23

*Note*: *CI* confidence interval

#### Causes

**Fig. 4:**
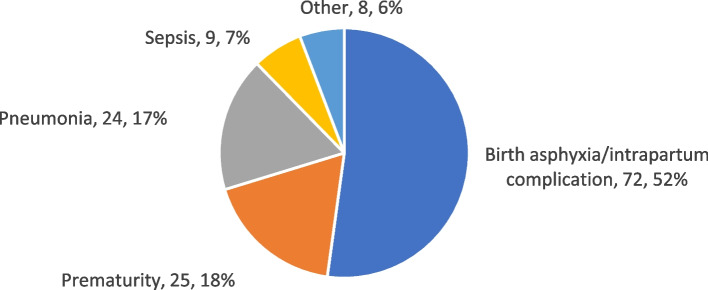
Causes of neonatal mortality – Areas A and B, October 2011–May 2015

The leading cause of neonatal mortality was intrapartum complication/birth asphyxia, present in 52% of neonatal deaths (Fig. 4). The second and third most frequent causes were complications of prematurity and pneumonia, present in 18% and 17% of deaths, respectively. Sepsis was present in 7% of cases, and other causes were present in 6% of cases.

#### Risk factors

**Fig. 5:**
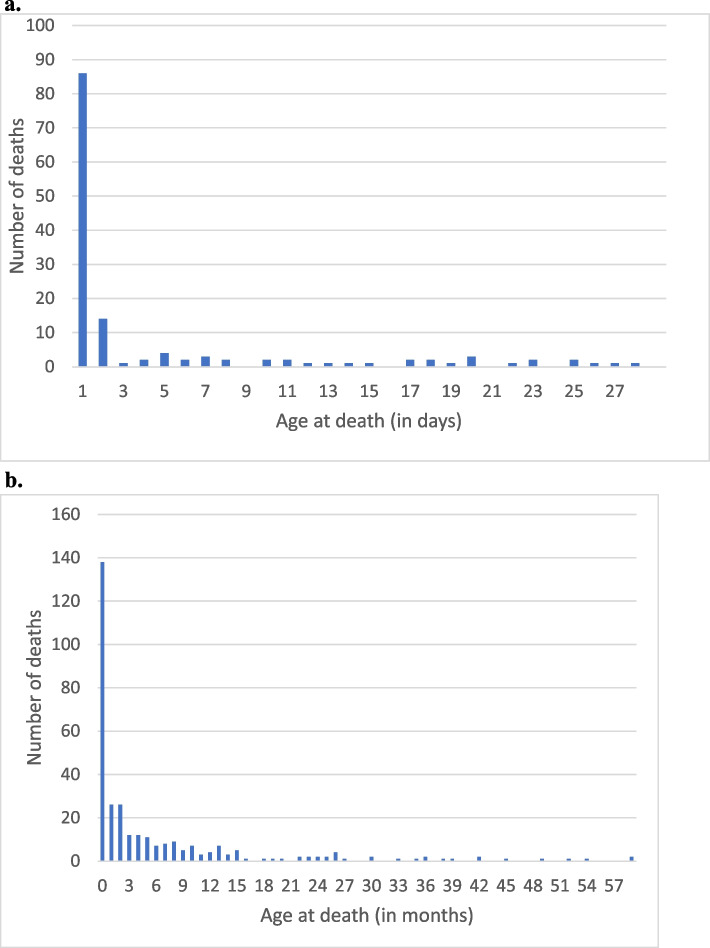
Number of deaths by age at death among children who died before 5 years of age in the Project Area, October 2011–May 2015. **a** Age at death (in days) among neonates. **b** Age at death (in months) among children 0- < 60 months of age in the Project Area, October 2011–May 2015

Two major risk factors for neonatal mortality were age and location of delivery. Across Area A and B communities, nearly two-thirds of neonatal deaths (61%, *n* = 84) occurred on the first day of life, and 81% (*n* = 112) of neonatal deaths occurred in the first week of life, as shown in Fig. 5a. Of the neonatal deaths from intrapartum complications/birth asphyxia, 79% occurred on the first day of life and 21% on the second day of life. 48% of neonates who died from complications of prematurity died on the first day of life, and the other deaths were spread throughout the neonatal period. Pneumonia deaths occurred more or less evenly throughout the neonatal period. Home births were associated with an increased risk of neonatal death: 80% of births took place at home, but 95% of neonates who died were born at home. In addition, 88% of neonatal deaths occurred at home (Appendix 2 in Fig. 10). Given our estimate that 80% of births took place at home (as discussed above under risk factors for maternal mortality), we estimate that the NNMR rate for newborns born at home during the period of Project implementation was 23.3 deaths per 1000 live births (133/5705 * 1000) compared to a NNMR of 3.5 deaths per 1000 live births (5/1426 * 1000) for newborns born in a facility, yielding an increased mortality risk for newborns born at home of 8.7 times that of the risk for newborn born in a facility (*p* = 0.00). Given the fact that two-thirds of neonatal deaths occurred on the first day of life, it is not surprising that 88% of neonatal deaths occurred in the home especially since facility-based care is so far away.

#### Changes over time in neonatal mortality

The NNMR in Area A communities fluctuated between 16 deaths per 1000 live births and 38 deaths per 1000 live births from year to year with no consistent trend (Table 2a). The NNMR in Years 1 and 2 was 18 deaths per 1000 live births, and in Years 3 and 4, it was 22 deaths per﻿ 1000 live births. The highest annual NNMR was 38 deaths per 1000 live births and occurred in Area A in Year 4. In Area B the NNMR increased from 14 deaths per 1000 live births to 29 deaths per 1000 live births in Years 3 and 4. These data suggest that the registration of neonatal deaths increased as duration of Project implementation increased, as we discuss further below.

Consolidating Years 1 and 2 and Years 3 and 4 in Area A demonstrated no clear trend (Table 2b). In the Area B communities, the NNMR increased from 16 deaths per 1000 live births to 21 deaths per 1000 live births between Year 3 and Year 4. None of the observed changes were statistically significant.

We also used LiST to estimate indirectly the decline in neonatal mortality in Project Area A on the basis of changes in the coverage of evidence-based maternal and neonatal health interventions. Appendix 5 provides further information about this. The LiST analysis estimates a neonatal mortality decline of 10% compared to a 5% projected decline in the absence of the Project, representing a net decline of 5%.

#### Comparisons between Areas A and B

As shown in Table 2b, in the Years 3 and 4 combined, the NNMR was slightly higher in Area A than in Area B, namely 22 deaths per 1000 live births versus 18 deaths per 1000 live births, but the difference is not statistically significant.

#### Comparisons with areas outside of the Project Area

The NNMR reported in the 2014/2015 national DHS was 17 deaths per 1000 live births [23]. The combined NNMR for Areas A and B in the final two years of the project was 20 deaths per 1000 live births, very similar to the national level.

#### The contribution of community birthing centers to the reduction in neonatal mortality

As mentioned previously, only 12.7% of the births included in the Vital Events Register took place in a Birthing Center. Thus, it is unlikely that the Birthing Centers contributed to a decline in the NNMR.

#### Registration of stillbirths

The Project registered stillbirths as well as live births. As we discuss in Appendix 4, in Area A the number of both stillbirths and live births registered increased over the four years of Project implementation. In Area B, the number of stillbirths registered increased markedly in Year 4 compared to Year 3 while the number of neonatal deaths registered remained essentially the same (see Appendix 4 in Table 13). These findings support the hypothesis that there was an under-registration of neonatal deaths initially, at least in Area A.

### Infant mortality

There were 214 deaths among live-born children who died before the first birthday (138 neonatal deaths and 76 deaths among children 1- < 12 months of age), yielding an infant mortality rate of 30 deaths per 1000 live births (data obtained from Fig. 5b). Further analysis of infant deaths is reported separately for neonatal deaths and deaths for the 1- < 60-month age group.

### Post-neonatal (1- < 60 month) mortality

There were a total of 176 deaths of children in the 1- < 60 month age group registered by the Project.

#### Level

Combining together all of the vital events data, we have an overall 1- < 60-month mortality rate of 25 deaths per 1000 live births (Table 3a and b).

**Table 3:** Number of births and deaths among children 1 < 60 months of age and corresponding mortality rates by Project Year and Project Area, October 2011–May 2015

Project year(s)		Communities											
		Area A communities				Area B communities				Area A and B communities combined			
		No. of live births registered	No. of 1- < 60 m deaths registered	1- < 60 m mortality rate	95% CI	No. of live births registered	No. of 1- < 60 m deaths registered	1- < 60 m mortality rate	95% CI	No. of live births registered	No. of 1- < 60 m deaths registered	1- < 60 m mortality rate	95% CI
a. Each year of Project activities													
1	Oct 2011- Sept 2012	1337	27	20	12, 28								
2	Oct 2012- Sept 2013	1352	45	33	23, 43								
3	Oct 2013- Sept 2014	1426	32	22	15, 30	1149	29	25	16, 35	2575	61	24	18, 30
4	Oct 2014- May 2015	906	23	25	15, 36	961	20	21	12, 30	1867	43	23	16, 30
b. Consolidated years and total													
1–2	Oct 2011- Sept 2013	2689	72	27	20, 33								
3–4	Oct 2013-May 2015	2332	55	24	17, 30	2110	49	23	17, 30	4442	105	24	19, 28
1–4	Oct 2011- May 2015	5021	127	29	24, 34	2110	49	23	17, 30	7131	176	25	21, 28

*Note*: *CI* confidence interval

#### Causes

**Fig. 6:**
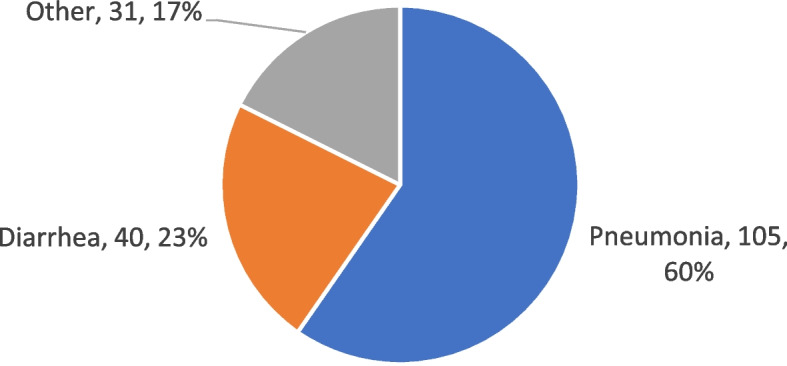
Causes of 1- < 60-month deaths in combined Areas A and B, October 2011–May 2015

As shown in Fig. 6, pneumonia is the cause of almost two-thirds (60%) of the 176 deaths in this age group, followed by diarrhea, accounting for about one-quarter (23%) of the deaths in this age group. There was no other single cause of death that was responsible for more than six deaths. Among the other very infrequent causes of death were congenital deformity, infection (other than pneumonia or sepsis), accident, prematurity, epilepsy, and meningitis.

#### Risk factors

##### Age

As Fig. 5b demonstrates, once an infant reached 1 month of age, the number of deaths that occur at each subsequent month of age was much smaller and declined progressively. Most (145 or 82%) of the 176 deaths during the period of 1- < 60-months of age occurred between 1 and 15 months of age, with the number steadily declining during that period. Only 18.3% of the deaths during the period of 1- < 60-months of age occurred after 15 months of age, and these were scattered out fairly evenly over the remaining 43-month period.

##### Location of death

As shown in Appendix 2 in Fig. 11, the great majority (83%) of deaths of children 1- < 60 months of age occurred at home.

#### Changes over time

The changes over time in mortality in this age group are shown in Table 3a and b. There were no discernable downward trends in mortality in this age group. In the full extended evaluation of the Project, we have carried out a more detailed analysis of the mortality trends for the 1- < 12 month and 12–59 month age groups for Area A and Area B separately [24]. There is a strong suggestion that there was an underreporting of deaths in the first year of Project operations in Area A since there is no other logical reason to account for the increase in the number of reported deaths in the second year of Project operations. Curamericas Global has seen similar increases in other CBIO projects that have been attributed to initial underreporting and improved vital events capture in the second year. The decline in 1- < 60-month mortality in Area B between the first and second year of Project operations there approaches but does not quite reach statistical significance.

If one were to accept the 1-<60-month mortality rate in Area A in Project Year 2 as a valid baseline rather than the first year rate, then one could make the case that in Area A there was a 1-<60-month mortality decline from 33 deaths per 1000 live births to 22–25 deaths per 1000 live births. This is not a statistically significant decline, however. Comparing the Area A 1- < 60-month mortality rate for Year 2 to Years 3 and 4 combined yields a decline in the rate from 33 deaths per 1000 live births to 24 deaths per 1000 live births. This difference does not reach statistical significance either. The mortality rates among children 12–59 months of age in Area A communities during the first three years of Project operations were between 8 deaths per 1000 live births and 10 deaths per 1000 live births each year, but during the final year of the Project, this mortality rate declined to only 2 deaths per 1000 live births. This comparison does in fact reach statistical significance when the data for PYs 1, 2, and 3 are combined and compared with the rate for PY 4 (see Appendix 1 Table 8 and Appendix 2 in Fig. 12).

#### Comparisons between Area A and Area B

As shown in Table 3, comparing the 1- < 60-mortality rates for Areas A and B during the same time periods reveals virtually no difference. All of the calculated rates are in the range of 22–29 deaths per 1000 live births.

### Under-5 (0- < 60-month) mortality

#### Level

As shown in Table 4, the overall under-5 mortality in the Project Area during the period of implementation was 44 deaths per 1000 live births.

**Table 4:** Number of births and deaths among children 0- < 60 months of age and corresponding under-5 mortality rates (U5MRs) by Project Year and Project Area, October 2011–May 2015

Project year(s)		Communities											
		Area A communities				Area B communities				Area A and B communities combined			
		No. of live births registered	No. of 0- < 60 m deaths registered	U5MR	95% CI	No. of live births registered	No. of 0- < 60 m deaths registered	U5MR	95% CI	No. of live births registered	No. of 0- < 60 m deaths registered	U5MR	95% CI
**a. Each year of Project activities**													
1	Oct 2011- Sept 2012	1337	49	37	26, 47								
2	Oct 2012- Sept 2013	1352	72	53	41, 66								
3	Oct 2013- Sept 2014	1426	49	34	25, 44	1149	47	41	29, 52	2575	96	37	30, 45
4	Oct 2014- May 2015	906	57	63	46, 80	961	40	42	28, 54	1867	97	52	41, 43
**b. Consolidated years and total**													
1–2	Oct 2011- Sept 2013	2689	121	45	37, 53								
3–4	Oct 2013- May 2015	2332	106	45	37, 54	2110	87	41	33, 50	4442	193	43	37, 50
1–4	Oct 2011- May 2015	5021	227	45	39, 51	2110	87	41	33, 50	7131	314	44	39, 49

*Note*: *CI* confidence interval

#### Causes

**Fig. 7:**
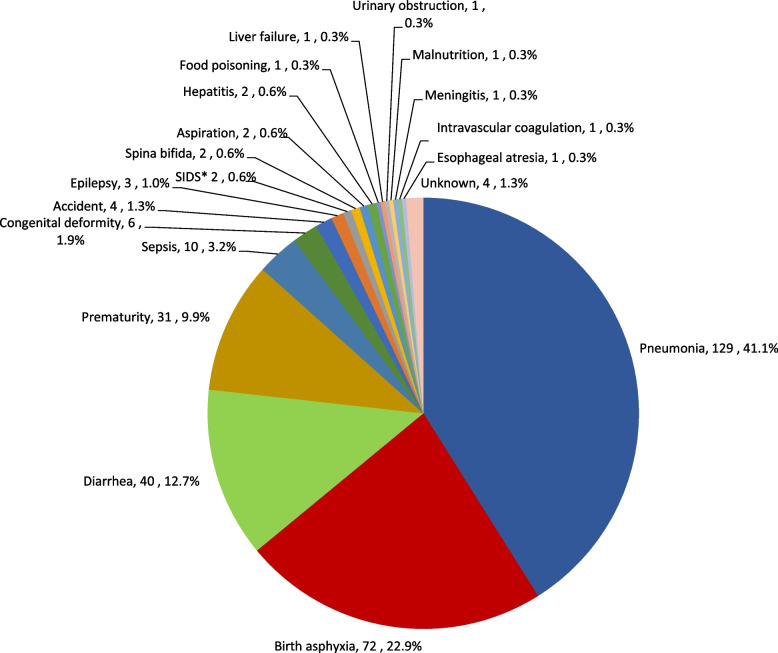
Causes of under-5 mortality for the entire Project Area, October 2011–May 2015. *SIDS: Sudden infant death syndrome

**Fig. 8:**
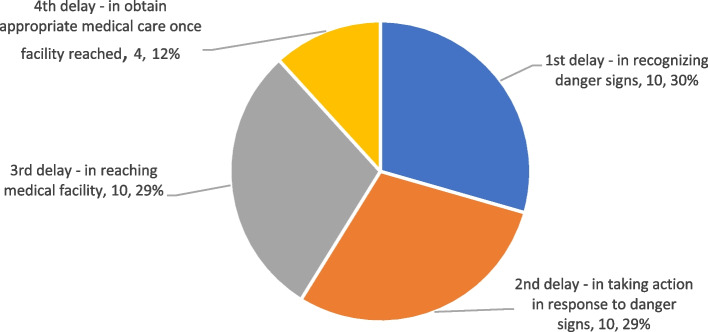
“Delays” that led to maternal death for both Areas combined, October 2011 – May 2015

**Fig. 9:**
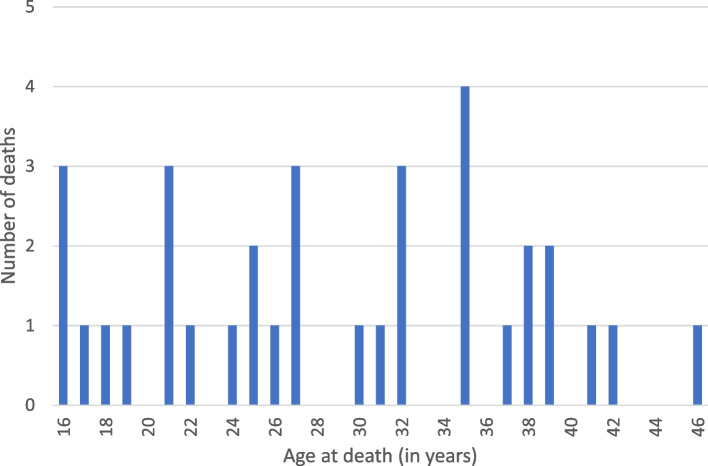
Number of deaths by age at death for maternal deaths, 2011–2015

**Fig. 10:**
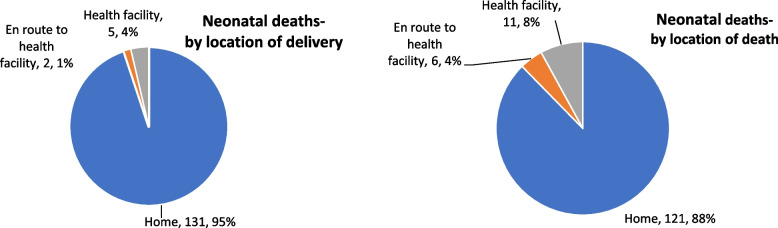
Location of birth and death for neonatal deaths – communities of both Areas combined, October 2011–May 2015

**Fig. 11:**
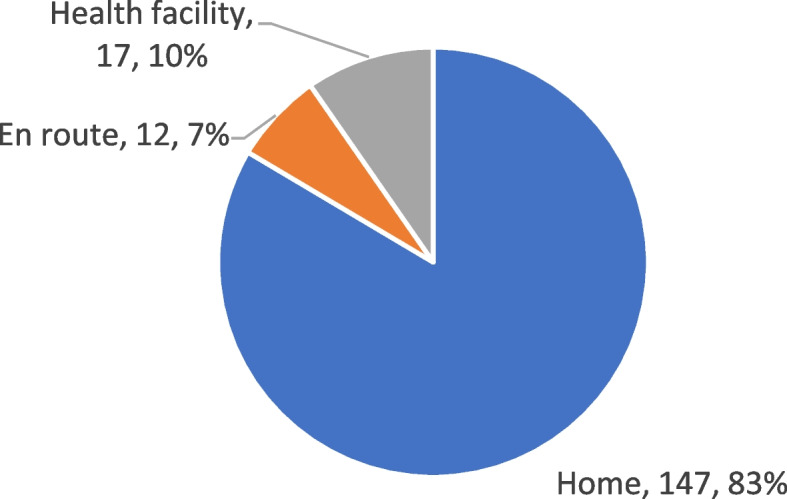
Location of death of children 1- < 60 months of age – communities of both Areas, October 2011–May 2015

**Fig. 12:**
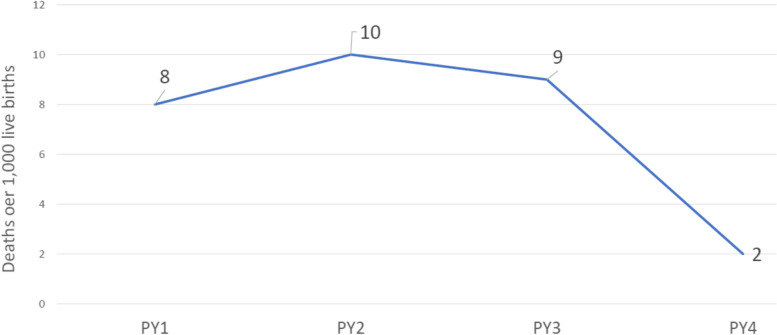
Trends in 12–59-month mortality in Area A, Project Years 1–4

Combining the causes of neonatal and 1- < 60-month deaths, pneumonia, responsible for 41% of deaths, was by far the leading cause of death. The second leading cause, responsible for 23% of deaths, was intrapartum complications/birth asphyxia. Diarrhea was the third leading cause (13% of deaths) followed by complications of prematurity (10%). These four causes represented 87% of all under-5 deaths. No other specific cause was responsible for more than 3% of under-5 deaths (see Fig. 7).

#### Risk factors

As shown in Fig. 5a and b, there was a marked concentration of under-5 deaths in the first month of life, and even within the first month of life, there was a marked concentration of deaths on the first day of life. 44% of the under-5 deaths occurred during the first month of life and, as previously mentioned, 62% of the neonatal deaths occurred on the first day of life.

##### Delays in seeking and obtaining care

As shown in Appendix 1 in Table 7, the most common of the four delays in seeking care for children in Area A who died before the age of 5 was in recognizing danger signs. This was the assigned delay for 42% of the deaths. The second delay, taking action in response to danger signs, and the fourth delay, obtaining appropriate medical care once the facility had been reached, were each determined to be the principal delay for approximately one-quarter of the under-5 deaths while delay in reaching the facility, present in 7% of the cases, was the least common.

#### Changes over time

As shown in Table 4, there was no evidence of a decline in under-5 mortality in Area A or Area B or in both areas combined. Just as we noted earlier for neonatal deaths, there is also a suggestion that there was an under-registration in Area A in the number of deaths during the first year of the Project, and, in fact, during the first three years of the Project since in Area A the under-5 mortality gradually increased from 37 deaths per 1000 live births in Year 1 to 63 deaths per 100,000 live births in Year 4. 

We used LiST to estimate indirectly the number of lives saved in Project Area on the basis of changes in the coverage of evidence-based child survival interventions. Appendix 5 provides further information about this. This analysis estimated an under-5 mortality decline of 24% compared to a 2% historical decline, yielding a net decline of 22% attributable to the Project,

#### Comparisons between Area A and Area B and with areas outside of the Project Area

The U5MRs in Areas A and B were essentially the same in Years 3 and 4: in Year 3, 34 deaths per 1000 live births in Area A versus 37 deaths per 1000 live births in Area B, and in Year 4, 63 deaths per 1000 live births in Area A versus 52 deaths per 1000 live births in Area B). Combining the data for these two years yields mortality rates of 45 deaths per 1000 live births in Area A and 43 deaths per 1000 live births in Area B. Thus, our hypothesis that the Project would have a greater mortality impact in Area A than in Area B is not supported. However, these mortality rates are 29% lower than the U5MR of 62 deaths per 1000 live births reported by the 2014/2015 DHS for the Department of Huehuetenango [23].

The national U5MR reported in the 2014/2015 DHS was 35 deaths per 1000 live births [23]. The combined U5MR for Areas A and B in the final two years of the project was 43 deaths per 1000 live births, 23% higher than the U5MR reported in the 2014/2015 national DHS.

## Discussion

This paper provides an in-depth assessment of levels, causes, risk factors, changes during the period of Project implementation, and comparisons with a quasi-control area (Area B) as well as with the Department of Huehuetenango for the mortality of mothers and their offspring in an isolated mountainous area of rural Guatemala inhabited by indigenous Maya people who have limited access to modern health services. The levels of mortality for mothers and under-5 children are on par with those of poor African countries such as Tanzania, Senegal, Uganda, and Zimbabwe, and the levels of mortality are among the highest in the Western hemisphere. For the four years of Project implementation, the MMR was 477 deaths per 100,000 live births, and the U5MR was 44 deaths per 1000 live births. From the standpoint of equity and public health, the Project Area and other areas similar to it in Guatemala should be seen as areas of priority for investments in strengthening health services. Instead, unfortunately, during the final year of the Project (2014), the government abandoned its program of outreach services in the Project Area and other underserved populations like it throughout the country. Having accurate data regarding the health status of underserved populations in Guatemala and elsewhere is a first step toward addressing these health inequities rather than allowing the actual health concerns of these underserved populations to be lost by merging the data about them into aggregated national data.

Our assessment also provides insight about the causes and underlying risk factors for maternal and under-5 mortality. For mothers, postpartum hemorrhage was the cause of 8 out of 10 maternal deaths, and risk of a maternal death was 8 times greater for women who gave birth at home compared to mothers who gave birth in a facility. For under-5 children, pneumonia was far and away the leading cause of death, responsible for 41% of the deaths. Intrapartum complications/birth asphyxia was responsible for one-quarter of the deaths, following by diarrhea and prematurity, each responsible for 1 in 10 deaths. The risk of a child death was markedly increased during the first month of life and especially on the first day of life: 44% of under-five deaths occurred during the neonatal period – the first 28 days of life – and 62% of neonatal deaths occurred on the first day of life.

Understanding the causes of death and the major risk factors for death using local data is a fundamental premise of the CBIO+ Approach, and the value of a mortality assessment for program planning and implementation is readily apparent on the basis of the findings presented here. For instance, the Project staff did recognize that the second leading cause of under-5 mortality was intrapartum complications/birth asphyxia, and this led to strengthened efforts for promoting deliveries at Community Birthing Centers*.*

Unfortunately, the full analysis of these data was not available until the end of the Project. Their utility for planning the next phase of Project activities was also limited since Curamericas/Guatemala did not have the financial resources to continue Project activities at the same level as was possible during the Project. However, the annual review of these data did underscore the importance of Birthing Centers for reducing maternal deaths from postpartum hemorrhage and neonatal deaths from intrapartum complications/asphyxia, giving a greater emphasis to the plans for the additional Birthing Centers that were later added in the Project Area as well as stocking the Centers with oxytocin, which was provided by Medicines for Humanity. The CBIO+ Approach, when fully implemented, requires iterations of 3-5-year cycles that begin with a community diagnosis based on a determination of local epidemiological priorities together with community-defined priorities. This is then followed by the development of a program plan based on available resources, implementation for 3–5 years of a program based on these priorities, and again a reiteration of the cycle beginning with a community health re-diagnosis [10]. Our paper provides strong evidence for the utility of a mortality assessment as a key element of the CBIO+ Approach.

While we consider the vital events registered by the Project in collaboration with the community to be relatively complete, we suspect that there was an initial under-registration of deaths, particularly in the first years of Project implementation, so the true baseline levels of mortality are quite likely to be even higher than what the Project documented. If so, this led to an under-estimate in mortality decline over the life of the Project. However, LiST, used to estimate mortality decline indirectly on the basis of changes in coverage of evidence-based interventions, suggests a net decline relative to ongoing changes in the absence of the Project of 12% in maternal mortality, 5% in neonatal mortality, and 22% in under-5 mortality. In spite of the suspected under-registration of maternal deaths at baseline, there is still evidence of a notable decline in maternal mortality in Area A between Years 2 and 4 and also a lower MMR in Area A than in Area B in Years 3 and 4 of the Project. Although there was not any evidence from the Vital Events Register of a decline in neonatal mortality, the data from the Vital Events Register for Area A did reveal a decline in the 1- < 60-month mortality rate and in particular the 12- < 60-month mortality rate. However, none of these changes reached statistical significance except for the comparison of 12–59-month mortality in Area A for Years 1–3, when it was 9 deaths per 1000 live births, and Year 4 when it was 2 deaths per 1000 live births, a difference that is statistically significant. Given the demonstrated role of undernutrition as a cause of approximately one-half of under-5 mortality in LMICs [25], the improvements in the nutritional status of children in the Project Area that we noted in Paper 4 provide strong indirect support, along with the improvements in coverage of key child survival interventions that we noted in Paper 3 [3], that the Project did have more of a mortality impact than were demonstrated by the analysis of data from the Vital Events Register.

### Maternal mortality

The MMR observed in the Project Area of 477 deaths per 100,000 live births is more than 5 times higher than the national levels reported by UNICEF [26] and Every Mother Counts [27] of 95 deaths per 100,000 live births and 73 deaths per 100,000 live births, respectively. The 2014–2015 DHS reported a somewhat higher national MMR of 140 deaths per 100,000 live births for the six years preceding the survey [23], but the MMR in the Project Area was still 3.4 times higher than this. The Department of Huehuetenango reported an MMR of 226 deaths per 100,000 live births in 2011, again only about one-half of that observed in the Project Area – but this includes the city of Huehuetenango and approximately one-third of the population of the Department that is non-Indigenous. The MMRs observed in the Project Area would have likely been even higher were it not for the efforts made by the Project to (1) train *comadronas*, traditional midwives who provide home deliveries, discussed in Papers 1 [2] and 6 in this series; (2) educate pregnant women and their families to recognize and seek care promptly at a facility when women develop a danger sign; and (3) develop and promote the use of Community Birthing Centers, where the *comadronas* can also participate*.*

In assessing maternal mortality results, we observed a strong relationship between the location of delivery and maternal mortality. Delays in obtaining care also influenced maternal mortality. All but two of the 34 maternal deaths in the combined set of communities in Areas A and B (*n* = 32, 94%) were associated with home deliveries, and hemorrhage was the cause of death in 82% (*n* = 28) of the cases. Home deliveries were associated with an eight-fold increased risk in mortality. This illustrates how location of delivery and time to obtaining emergency care are critical factors in ensuring maternal survival. Over one-quarter of maternal deaths occurred en route to a health facility, correlating with the large percentage of "third" delays. The closest referral hospital is in the city of Huehuetenango, a four-hour drive with most of it over difficult unpaved mountain roads. This contributed to the number of respondents who cited the "third" delay, related to transportation, when reporting maternal deaths.

The MMR did decline in the Project Area, and there was other progress to support this, as we presented in Paper 3. There were marked increases in knowledge of danger signs during pregnancy and delivery. There were also marked increases in antenatal care utilization, awareness of danger signs, utilization of facilities for delivery, percentage of deliveries in which there was active management of the third stage of labor, and use of trained attendants, including an increased utilization of Community Birthing Centers. Finally, the percentage of births that were delivered by cesarean section during the Project’s final two years was statistically significantly greater in Area A than in Area B (8.7% versus 2.3%, *p* = 0.001). (Unfortunately, there was no baseline measure of the prevalence of cesarean section in either Area, but it seems reasonable to assume the baseline levels in the two Areas were similar). Our hypothesis that there would be a larger mortality reduction in Area A due to longer exposure to interventions was borne out for maternal mortality.

### Neonatal mortality

Our mortality assessment highlights the importance of neonatal mortality, during which time 44% of under-5 deaths occurred, as a major component of under-5 mortality together with the greatly increased risk of death during the first day of life, at which time 61% of neonatal deaths occurred. Importantly, home deliveries were associated with an eight-fold increased risk of neonatal mortality. The Project did its best to identity stillbirths and to make a clear distinction between stillbirths and live-born babies who died soon after birth. As shown in Appendix 4, we carried out a separate analysis of the number of stillbirths reported in the Vital Events Register for each year to see if there were any notable changes over time as the Project continued to emphasize the need to report stillbirths and live births accurately. It appears that there was an increasing detection of both stillbirths and neonatal deaths in Area A and a decreasing misclassification of true neonatal deaths as stillbirths in Years 3 and 4 in Area B. Nonetheless, it is important to emphasize that under-registration of neonatal deaths is a recognized problem because of the reluctance of mothers to share this information and because of the confusion that exists in distinguishing a death of a newborn who dies shortly after birth (and therefore should be classified a neonatal death) from the death of an infant who is born without any signs of life (and therefore should classified as a stillbirth) [28].

Our vital events data showed a marked increase in neonatal mortality in Area A in Year 2 of the Project. This suggests that there may have been an underreporting of neonatal deaths during Year 1 since there was no other apparent reason for the increase. The net effect was that the vital events registration system revealed no apparent decline in neonatal mortality. There are several other potential explanations for why we observed no decline in neonatal mortality: the loss of the preventive and curative services of the MSPAS Extension of Coverage Program, which provided primary health care, and the increase in the local cost of transportation; increased poverty due to the loss of remittances from men working in the US; and the effects of the Guatemalan socio-political crisis, which caused deterioration of local health services. Of course, these contextual effects would have affected maternal and 1- < 60-month mortality also, but since the reductions in maternal and 1- < 60-month mortality were more pronounced, we were able to observe a decline that might have been even greater in the absence of these contextual challenges.

### 1- < 60-month mortality

Only 17% of deaths in this age group occurred after 15 months of age. Pneumonia, responsible for two-thirds of the deaths, was far and away the leading cause of death in this age group. The number of deaths declined as age increased. According to data from the vital events registry, there was no decline in the 1- < 60-month mortality, though the 12- < 60-month mortality did show a significant decline.

### 0- < 60-month mortality

Apart from the findings mentioned above for neonatal and 1- < 60-month mortality, we were able to estimate a net decline in under-5 mortality of 22% using LiST. Given the challenges noted in initially achieving complete registration of vital events leading to what we consider to be underestimates of mortality rates during the initial stages of Project implementation, the LiST estimates seem reasonable. This is because marked improvements were documented in the coverage of evidence-based child-survival interventions, as described in detail in Paper 3 of this series [3].

### Constraints on mortality impact and its assessment

The lack of stronger evidence for an impact on maternal, neonatal, and 1- < 60-month mortality can be attributed to several factors. First, four years of Project implementation in Area A and only two years in Area B are both short times to expect to achieve a notable decline in mortality in such a challenging context. Second, even though the Project staff made a valiant attempt to encourage community members and especially Care Group Volunteers, who were in touch with all households, to report all vital events, this is not easily achieved – especially with a short start-up time before Project interventions began prior to the establishment of an accurate vital events registration system and stable measures of baseline mortality levels. Third, the abrupt cancellation of the Extension of Coverage Program (PEC) by the government in the fall of 2014, as discussed further in Paper 1 in this series [2], made it impossible to continue local services that had been provided by the PEC nurses: immunizations, family planning, antenatal care, treatment of acute childhood illness, and nutritional monitoring. This probably limited, to some degree, the Project’s impact on mortality since referral for these services, which the Project promoted, was no longer possible.

Finally, the Project did not have at its disposal the full armamentarium of interventions that are recommended by the World Health Organization (WHO) that could have saved lives. This was because the MSPAS had not approved the use of these interventions in Guatemala. These interventions include the use of oral misoprostol after a home birth to reduce the risk of postpartum hemorrhage and integrated community case management (iCCM) of childhood illness. Further, zinc, as recommended by WHO, was not available for the treatment of diarrhea. Finally, the Project did not have the capacity to train its staff to implement home-based neonatal care, which requires, among other things, frequent home visits to promote and deliver essential newborn care. Of note as well is that Guatemala abandoned its earlier national program of community health workers who could have been trained to implement these interventions [29].

### Programmatic implications

Efforts to reduce maternal mortality and under-5 mortality must focus on addressing the leading causes of death, which in the Project Area were postpartum hemorrhage, intrapartum complications/birth asphyxia, pneumonia, and diarrhea. Raising community and family awareness of danger signs associated with these causes of death is essential. Potential interventions include additional health promotion activities that focus on facility delivery and the use of Birthing Centers; care of the newborn, particularly during the first week of life; prompt care-seeking and attention to childhood illness, particularly pneumonia and diarrheal disease; and the ready availability of appropriate clinical care, including iCCM [30]. Our vital events data also support the need for the WHO-recommended strategy of advance oral misoprostol distribution to mothers who intend to deliver at home, so that it can be taken immediately after birth to reduce the risk of postpartum hemorrhage [31] as well as the WHO-recommended strategy of training community-based workers to provide iCCM. Unfortunately, during the time of Project implementation, these interventions had not been approved by the MSPAS. Continued efforts to mitigate geographical and transportation barriers and reduce the time required to reach appropriate medical care will also be necessary.

Our Project data reinforce the case for Community Birthing Centers since virtually all maternal deaths and neonatal deaths occurred when the mother delivered at home. The Birthing Centers improve access to clean, safe, and high-quality delivery services in a culturally friendly way. The Birthing Centers also provide services that respond to the leading causes of maternal and neonatal deaths. There, skilled health workers provide management of the third stage of labor (including provision of oxytocin to mothers as soon as the baby is delivered), and they are ready to manage pregnancy complications including postpartum hemorrhage, resuscitation of babies who are not breathing after birth, and a prompt referral if the complication cannot be appropriately handled at the Birthing Center*.* There remains a need for continued maternal care education to women and their families – especially as it relates to the recognition of danger signs, provision of accessible transportation services for medical emergencies, and expansion of the Birthing Centers. Paper 6 in this series provides further details about the quality of care at the Birthing Centers and the management of complications. Also, during the period of Project implementation, assistance from the NGO *Medicinas para la Humanidad* made it possible to establish small self-sustaining pharmacies with a rotating drug fund at the Birthing Centers for the auxiliary nurses working there to treat cases of childhood pneumonia, diarrhea, and other acute illnesses.

### Mortality assessments of local programs carried out elsewhere

The approach used here, carrying out a mortality assessment based on prospective registration of vital events through routine systematic home visitation to guide and assess a local health program, has been utilized infrequently. Such assessments using the CBIO approach have been reported from Bolivia [32, 33], and assessments using the Care Group approach have been reported from Mozambique [34]. In the latter case, the accuracy of the under-5 mortality assessment was verified using an independent assessment of changes in under-5 mortality obtained from retrospective maternal birth histories [34]. The Society for Education, Action and Research (SEARCH) in Gadchiroli, India, has used the registration of vital events through routine monthly systematic home visits by CHWs to assess the mortality impact of its interventions on childhood pneumonia [35] and home-based neonatal care [36, 37]. There are examples from Haiti [38, 39] and Mali [40] in which programs using some CBIO principles (including routine systematic home visitation) have assessed their mortality impact by measuring mortality changes through repeated retrospective surveys. LiST has been used to estimate mortality reductions of child survival projects using the Care Group approach compared to the child survival projects using other approaches [18, 41]. The external evaluator of the Curamericas Global child survival project in Liberia implementing CBIO and Care Groups used LiST to estimate a 63% decline in under-5 mortality [42].

### Limitations

The prospective collection of vital events through frequent home visitation is a core element of the CBIO Approach. However, ensuring that all vital events are being registered is a challenge. We had no quality control system to verify the completeness of registration. The development of such a system is a critical need to refine the mortality assessment of the CBIO Approach. One feasible way to do this is used by the Sample Registration System in India [43] and by SEARCH [36] in India, which entails the hiring of someone to visit every home every 6 months to record any vital events that had taken place since the previous visit and then cross tabulating the findings from both registration methods. An alternative approach is now being implemented by Curamericas Global in Kenya and Haiti to carry out an annual household census and, at that time, independently register all births and deaths that had taken place in the previous year.

Another limitation is that there may have been inconsistencies and inaccuracies in classifying the cause of death, in assigning the most appropriate delay that contributed to the death, and in differentiating stillbirths from neonatal deaths. Additionally, verbal autopsies to determine cause of death are affected by the degree of trust the respondent has in the interviewer, recall error, and reporter error as a result of shame or guilt. This means that the families interviewed may have, for instance, reported that they did not recognize danger signs in order to absolve themselves of responsibility. The Project also lacked a standard method for determining which delay was the most important in contributing to the death, and we are not aware that such a method exists.

Of note, as well, is the fact that our vital events registration system did not record any maternal deaths related to abortion. Globally, abortion causes 8–13% of maternal deaths but is often under-reported and difficult to detect, especially in settings in which most deaths occur at home [44].

### The value of mortality assessment

In spite of these limitations, the findings from our mortality assessment are of great importance for the Project Area, for rural Guatemala more broadly, and for the advancement of the CBIO+ Approach. With this information from the mortality assessment in hand, changes in the program can be made to enable it to continue to reduce the number of deaths from readily preventable or treatable causes. These findings are important, not only for the Project, but also for the community.; They can serve as a powerful motivator to the community to strengthen their own addressing the leading causes of death among mothers and their offspring.

The use of vital events data to provide up-to-date measures of mortality of mothers and children at local levels is an emerging priority for programs throughout the world. The method that we use here represents an important advance beyond the traditional approach of relying on national DHS surveys whose data are aggregated over larger geographic areas and already out of date by the time they become available because these estimates refer to a recall period spanning 5–10 years in the past. Through the methods presented here, it is possible to produce reasonably accurate mortality assessments on a quarterly basis for a relatively small population and to use these findings to guide program efforts in collaboration with the community. There have been ongoing efforts for at least four decades by some pioneering non-governmental programs to register vital events [32, 33, 35, 45] as well as more recent efforts to incorporate vital events registration and cause of death assessments into the roles of community health workers [46–50]. A field manual for recording and tabulating vital events data arising from routine systematic home visitation is available to facilitate the mortality assessment process with local data [51]. This is a field of endeavor in which the development and incorporation of digital tools and other mHealth applications could be fruitful.

## Conclusion

The mortality assessment described here is an example of what can be achieved by a local program with minimal external technical support. As such, this mortality assessment can serve as a guide for other pioneering community-based primary health care programs. With continued use and experience, mortality assessments can become standard practice in district-level programs in areas where the risk of death from readily preventable or treatable conditions remains excessive.

## Figures and Tables

**Fig. 13:**
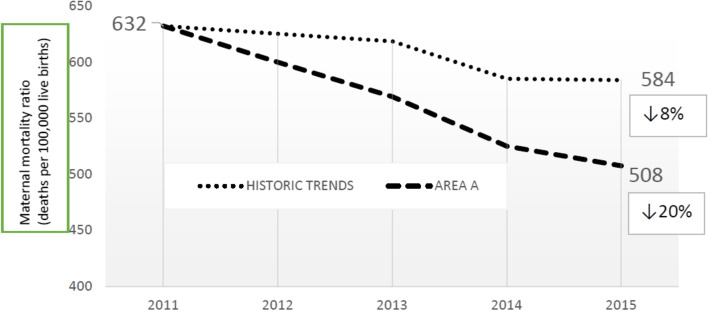
Estimate of decline in maternal mortality in Area A, 2011 to 2015 using the Lives Saved Tool, compared to estimated historical decline

**Fig. 14:**
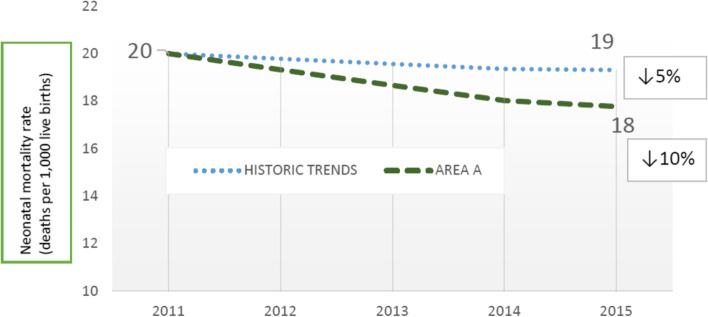
Estimate of decline in neonatal mortality in Area A, 2011 to 2015 using the Lives Saved Tool, compared to estimated historical decline

**Fig. 15:**
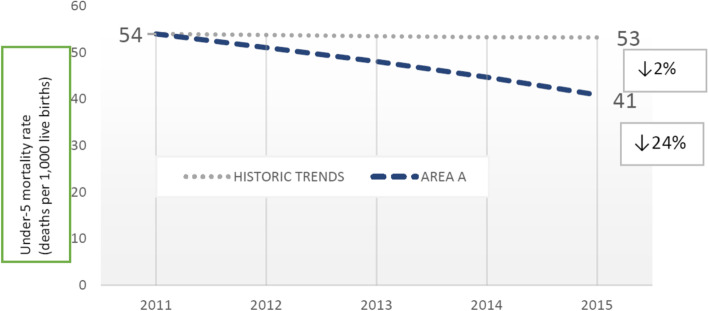
Estimate of decline in 0- < 60 month mortality in Area A, 2011 to 2015, using the Lives Saved Tool, compared to estimated historical decline

## Data Availability

All of the Project reports, de-identified data, as well as publications about the Expanded CBIO+ Approach cited in this article are available from the corresponding author on request.

## Appendix 1

### Additional tables

Tables 5, 6, 7, 8

**Table 5:** Causes of maternal deaths from hemorrhage

Cause of hemorrhage	Area A communities (Oct 2011-May 2015)		Area B communities (Oct 2013-May 2015)		Areas A and B combined	
	N	%	N	%	N	%
Retained placenta	14	74%	7	78%	21	75%
Uterine atony	4	21%	1	11%	5	18%
Uterine rupture	1	5%	1	11%	2	7%
Total	19	100%	9	100%	28	100%

**Table 6:** Causes of non-maternal deaths among women of reproductive age

Cause	Number
Cancer (2 from leukemia, 1 from uterine cancer, 2 unknown type)	5
Diabetes	4
Pneumonia	4
Suicide	4
Alcoholism/cirrhosis	3
Myocardial infarction	3
Epilepsy	2
Infection (otherwise unspecified)	1
Fall	1
Renal failure	1
Vomiting/diarrhea	1
Total	29

**Table 7:** Changes in the “delays” in seeking care for under-5 children in Area A who died between October 2011 and May 2015

Delay	Years 1–3 (October 2011–September 2014)		Year 4 (October 2014–May 2015)		Total	
	N	%	N	%	N	%
1-In recognizing danger signs	58	34.3	34	65.4	92	41.6
2-In taking action in response to danger signs	60	35.5	6	11.5	66	29.9
3-In reaching a medical facility	13	7.7	3	5.8	16	7.2
4-In obtaining appropriate medical care once facility reached	38	22.5	9	17.3	47	21.3
Total	169	100.0	32	100.0	221	100.0

Note: No “delays” were assigned for 6 deaths

Chi-square (3 df) 17.57, *p* = 0.0005 (comparing Years 1–3 with Year 4).

**Table 8:** Comparison of 12- < 60-month mortality rates for Years 1–3 with Year 4 in Area A

Year	No. of live births registered	No. of 12- < 60 m deaths registered	12- < 60-month mortality rate	95% confidence interval
1–3 (Oct 2011-Sept 2014)	4115	37	9	6, 12
Year 4 (Oct 2014- May 2015	906	2	2	0, 5

Chi-square (3 df) 4.43, *p* = 0.035.

## Appendix 2

### Additional figures

Figures 8, 9, 10, 11, 12.

## Appendix 3

### Analysis of the effectiveness of Community Birthing Centers (*Casas Maternas Rurales)* in reducing maternal and neonatal mortality

*Casas Maternas Rurales* are Community Birthing centers that have been established by Curamericas/Guatemala in collaboration with local communities, and the communities that have taken responsibility for operating a given Birthing Center in collaboration with Curamericas/Guatemala are referred to as partner communities. Paper 1 [2] and Paper 6 [5] provide more information about these Birthing Centers. Unfortunately, our Vital Events Registry did not provide information on the place of birth in the birth registry. We did, however, obtain information about the details of the location of birth at the time of the baseline and endline knowledge, practice, and coverage (KPC) surveys in Areas A and B, as we reported in Paper 3 [3]. At baseline, during the previous two years 3.0% of births in Area A and 1.7% in Area B took place at a Birthing Center*,* and at endline, there was a modest increase to 11.0 and 3.0% in Area A and Area B, respectively (Appendix 3 in Table 9).

**Table 9:** Location of births at Project Baseline (2011) and Endline (2015) in Areas A and B (based on KPC survey data)

Location	Area A		Area B	
	Baseline	Endline	Baseline	Endline
**In a health facility**	16.4% (49/299)	29.3% (88/300)	6.7% (20/300)	13.0% (39/261)
** Community Birthing Center**	3.0% (9/299)	11.0% (33/300)	1.7% (5/300)	3.0% (9/300)
** Other health facility**	13.4% (40/299)	18.3% (55/300)	5.0% (15/300)	10.0% (30/300)
**Home**				
**At home by** ***comadrona***	83.3% (247/299)	70.7% (212/300)	93.3% (280/300)	87.0% (261/300)
**Total**	100.0% (299/299)	100.0% (300/300)	100.0% (300/300)	100.0% (300/300)

When the Project began its operations in October 2011, only one Birthing Center was operating, in Calhuitz in Area A. In PY 2, a second Birthing Center began operating in Santo Domingo, and in PY 3, a third Birthing Center began operating in Tuzlaj Coya. Although the time periods for calculating the number of births in the Birthing Center and in the Project Area did not overlap precisely, they are roughly analogous. The tabulations for Birthing Centers are for calendar years (January to December) while the tabulations for the entire Project Area are for the 12-month period from October through September. As shown in Appendix 3 in Table 10, 12.7% of the 7131 births during the period of Project implementation took place in a Birthing Center.

**Table 10:** Number of births occurring at Community Birthing Centers and total number of births in the Project Area, October 2011–May 2015

Number of births at Community Birthing Centers (***Casas Maternas Rurales***) according to Birthing Center records						Total number of births in Project Areas A and B (combined)		Percentage of births in Project Area taking place in a Community Birthing Center
Period		Calhuitz (Area A)	Santo Domingo (Area A)	Tuzlaj Coya (Area B)	Total	Period	Total	
1	Oct 2011- Sept 2012	95	0	0	95	Oct 2011-Jan 2012 (Area A only)	1337	7.1%
2	Oct 2012- Sept 2013	133	57	0	190	Oct 2012-Jan 2013 (Area A only)	1352	14.1%
3	Oct 2013- Sept 2014	127	60	59	246	Oct 2013-Jan 2014 (Areas A and B combined)	2575	9.6%
4	Oct 2014- May 2015	106	76	112	294	Oct 2014-May 2015 (Areas A and B combined)	1867	15.7%
Total		542	193	171	906		7131	12.7%

It is not realistic to expect that during the period of Project implementation the Birthing Centers themselves would have been responsible for a decline in maternal and/or neonatal mortality for the entire Project Area, but it is important to ask if those women and their infants who gave birth in a Birthing Center had a lower risk of death than those who gave birth elsewhere. We address this question indirectly by analyzing the maternal and neonatal mortality in the communities that surround the three Birthing Centers during the time of Project implementation.

### Maternal mortality

To estimate the contribution of the three Birthing Centers to the reduction of maternal mortality, we disaggregated the maternal mortality data into births and deaths that took place in partner and non-partner communities. The three Birthing Centers had a total of 26 partner communities, making up three “micro-regions.” These communities, all surrounding one of the Birthing Centers, built and then supported the functioning of the Birthing Center. 13.2% of the births recorded during the Project occurred to a mother residing in one of these communities. The data for these communities are compared with those for 154 non-partner communities where 86.5% of the births took place. As shown in Appendix 3 in Table 11, in the 26 partner communities the MMR declined from 508 deaths per 100,000 live births in PY 1 to 0 deaths per 100,000 live births in PY 4; in the 154 non-partner communities, the MMR declined from 526 deaths per 100,000 live births in PY1 to 491 deaths per 100,000 live births in PY 4. If the data for all four years of the Project are combined, the MMR in the Birthing Center partner communities was 318 deaths per 100,000 live births compared to 501 deaths per 100,000 live births for the non-partner communities. Given the small number of maternal deaths (3 in the partner communities and 31 in the non-partner communities), the 95% confidence intervals are extremely large, and the difference is not statistically significant. Nonetheless, the data are suggestive of a possible effect of the Birthing Centers on reducing maternal mortality.

**Table 11:** Yearly number of live births, maternal deaths, and maternal mortality ratios (MMRs) in partner communities of Community Birthing Centers (*Casas Maternas* Rurales) and non-partner communities, October 2011–May 2015

Project Year		26 ***Casa Materna Rural*** partner communities				154 non-partner communities			
		No. of live births	No. of maternal deaths	MMR	95% confidence interval	No. of live births	No. of maternal deaths	MMR	95% confidence interval
1^a^	Oct. 2011 - Sept. 2012	197	1	508	321, 681	1140	6	526	97. 956
2^a^	Oct. 2012 - Sept. 2013	234	1	427	1, 1282	1118	9	805	268, 1342
3^b^	Oct. 2013 - Sept. 2014	273	1	366	0, 1099	2302	8	348	102, 593
4^b^	Oct 2014 - May 2015	238	0	0	0, 0	1629	8	491	144, 838
Total	Oct 2011-May 2015	942	3	318	0, 686	6189	31	501	321, 681

^a^Area A communities only; ^b^Area A and Area B communities combined

### Neonatal mortality

In the 26 partner communities, there was a suggestion of a decline in the NNMR, and in the 154 non-partner communities there was no apparent trend over time in neonatal mortality nor was there any apparent difference in the NNMR between the partner and the non-partner communities (Appendix 3 in Table 12). If one collapses the years 2–4 for the partner communities, this yields a NNMR of 17.4 deaths per 1000 live births with 95% CI of 8 deaths per 1000 live births and 27 deaths per 1000 live births. The difference between this NNMR of 17.4 deaths per 1000 live births and the NNMR of 41 deaths per 1000 live births in PY1 is striking, but the difference is not statistically significant.

**Table 12:** Yearly number of live births, neonatal deaths, and neonatal mortality rates (NNMRs) in partner communities of Community Birthing Centers (*Casas Maternas* Rurales) and non-partner communities, 2011–2015

Project Year		26 ***Casa Materna Rural*** partner communities (from both Areas)				154 non-partner communities (from both Areas)			
		No. of live births	No. of neonatal deaths	NNMR	95% confidence interval	No. of live births	No. of neonatal deaths	NNMR	95% confidence interval
1^a^	Oct 2011-Sept 2012	197	8	41	12, 69	1140	14	12	6, 19
2^a^	Oct 2012- Sept 2013	234	3	13	0, 28	1118	24	21	13, 30
3^b^	Oct 2013- Sept 2014	273	4	15	0, 29	2302	31	13	9, 18
4^b^	Oct 2014- May 2015	238	6	25	5, 46	1629	48	29	21, 38
1–4^b^	Oct 2011- May 2015	942	21	22	13, 32	6189	117	19	15, 22

^a^Area A only; ^b^Areas A and B combined

## Appendix 4

### Analysis of Stillbirths

One of the hypotheses to explain the unexpected increase in neonatal mortality that our Vital Events Register recorded is that in the early years of the Project, some neonatal deaths were misclassified as stillbirths and, as the Project matured, the quality of the classification of deaths improved. As there were fewer neonatal deaths that were falsely classified as stillbirths, there was an increase in the neonatal death rate. As one attempt to assess the validity of this hypothesis, we examined the number of deaths that we classified as stillbirths and neonatal deaths by each year of the Project, as shown in Appendix 4 in Table 13. We adjusted the numbers for PY4 to reflect what would have been observed if the Project had been implemented for a full 12 months in PY4.

As shown in Appendix 4 in Table 13, in Area A the number of registered stillbirths doubled between PY1 and PY4 (from 21 to 54) as did the number of neonatal deaths (from 22 to 51). Thus, our hypothesis was not supported there. This finding is consistent with the interpretation that during the early years of Project implementation more stillbirths and neonatal deaths were being missed than in the later years.

In Area B, however, the number of stillbirths more than doubled from PY3 to PY4 (from 21 to 57) while the number of neonatal deaths remained similar (18 and 20). This then appears to support the hypothesis that more true neonatal deaths were classified as stillbirths in PY3 than in PY4. This would suggest that the NNMR calculated for Area B in PY3 was more artificially depressed than in PY4, and thus the increase observed in the NNMR between PY3 and PY4 was artifactual rather than a true increase.

Therefore, no definite conclusions can be drawn, but the most plausible explanation for the findings as they related to neonatal mortality is that in Area A, there was an increasing registration of both stillbirths and neonatal deaths each year with no evidence of misclassification, while in Area B there is a suggestion of misclassification of neonatal deaths in Year 3 and fewer neonatal deaths classified as stillbirths in Year 4.

**Table 13:** Stillbirths, neonatal deaths, and modified perinatal mortality in Areas A and B, October 2011–May 2015

Year		Area A Communities						Area B Communities					
		Number of live births	Number of still-births	Number of births (still + live)	Number of neonatal deaths	Total number of “perinatal”^a^ deaths (stillbirths + neonatal deaths)	Modified perinatal mortality rate^a^	Number of live births	Number of still-births	Number of births (still + live)	Number of neonatal deaths	Total number of “perinatal”^a^ deaths	Modified perinatal mortality rate^a^
PY1	Oct 2011-Sept 2012	1333	21	1354	22	43	31.8						
PY2	Oct 2012- Sept 2013	1352	58	1410	27	85	60.3						
PY3	Oct 2013- Sept 2014	1426	46	1472	17	63	42.8	1149	21	1170	18	39	33.3
PY4	Oct 2014- May 2015	906	36	942	34	70	74.3	961	38	999	20	58	58.0
PY4^b^	Oct 2014-Sept 2015	1359	54	1412	51	105	74.3	1442	57	1499	30	87	58.0

^a^We are calculating here a modified perinatal mortality rate, which is the number of stillbirths plus the number of neonatal deaths divided by the number of births (stillbirths plus live births) multiplied by 1000. The actual perinatal mortality rate uses only early neonatal deaths, which occur the first week of life)

^b^ Since PY4 lasted only 8 months, we have estimated the number of births and deaths that would have occurred during a 12-month period if the same average monthly number had continued for a full 12 months. This was done by multiplying the numbers of births and deaths by 1.5. The mortality rates remained the same

## Appendix 5

### Indirect estimate of maternal and under-5 mortality decline using LiST (the Lives Saved Tool)

We used the Lives Saved Tool (LiST) to estimate indirectly changes in the MMR, NNMR, and U5MR in Project Area A on the basis of changes in the coverage of evidence-based maternal and child health interventions. We excluded Area B from this analysis because of the short time period (20 months) of Project implementation there. We estimated the baseline MMR by calculating the MMR for the first two years of the Project. In the case of the U5MR, we elected to use the baseline rate observed in Year 2 of the Project.

LiST is a module within the Spectrum package with linkages for demography, fertility determinants, and HIV/AIDS interventions [13]. Details about the LiST methodology have been described elsewhere [52]. LiST estimates maternal, child, and pregnancy outcomes based upon changes in population-level coverage of interventions while considering the underlying health status, cause-specific mortality distributions, and the best available estimates of intervention effectiveness using a linear deterministic model. We used the coverage levels at the beginning and end of the Project for interventions that are included in Paper 3 in this series as shown in Appendix 5 Table 14 [3]. Estimates of the historical decline of mortality were obtained from the LiST data library based on national DHSs, Multiple Cluster Indicators Surveys, and other national household surveys. Further details regarding the actual calculations are available from the corresponding author upon request.

Appendix 5 Figs. 13, 14, and 15 in Appendix 5 show the estimated declines in mortality based on changes in the population coverage of evidence-based interventions in Area A between baseline in 2011 and endline in 2015. There is an estimated decline in maternal mortality of 20% compared to a historical decline of 8%, yielding a net decline of 12%. There is a 5% net decline in neonatal mortality and a 22% net decline in under-5 mortality.

**Table 14:** Baseline and endline KPC data from Area A used for the Lives Saved Tool calculations

Indicator	Baseline KPC (October 2011)	Endline KPC (June 2015)
At least 4 quality antenatal care checks during most recent pregnancy	13.4%	65.0%
Tetanus toxoid immunization during most recent pregnancy	63.2%	67.7%
Iron/folate for at least 90 days during most recent pregnancy	21.7%	64.3%
Percentage of children 0- < 24 months of age whose births were attended by skilled personnel (doctor, nurse, professional midwife)	15.4%	29.3%
Percentage of births that took place in a health facility (hospital, clinic, or Birthing Center)	16.4%	28.7%
Percentage of births receiving active management of the third stage of labor (AMTSL) during most recent delivery	9.4%	20.0%
Postpartum visit for the mother and newborn within 48 hours after delivery	22.4%	39.0%
Essential newborn care during most recent delivery	6.0%	39.0%
Current use of modern contraception	35.8%	34.0%
Birth interval < 24 m between previous 2 deliveries	32.7%	24.7%
Exclusive breastfeeding (children 0- < 6 months) in previous 24 hours	75.0%	82.0%
Proper infant and young child feeding (children 6- < 24 months of age)	53.0%	74.3%
Vitamin A supplementation for child 6- < 24 months of age in previous 6 months	79.1%	74.3%
Children with cough and rapid/difficult breathing in the 2 weeks prior to the interview	25.8%	20.7%
Appropriate care seeking for child with symptoms of pneumonia	26.1%	51.6%
Children with diarrhea episode in the 2 weeks preceding the interview	40.1%	34.3%
Use of oral rehydration therapy or a recommended home fluid during a diarrheal episode	28.3%	40.8%
Increased fluid intake during a diarrheal episode	7.5%	18.5%
Increased food intake during a diarrheal episode	0.0%	0.0%
Zinc treatment for diarrhea	6.7%	10.7%
Regular point-of-use water treatment	66.6%	97.7%
Safe water storage	11.7%	28.0%
Safe disposal of child’s feces the last time s/he/ defecated	43.1%	45.0%
Appropriate hand washing station in the home (with water, soap, and convenient place to store the soap)	2.3%	44.7%
Hand washing at the 4 critical times: after defecating, before preparing food, after cleaning a child, and before feeding a child	1.3%	34.0%
Measles immunization in children 12- < 24 months of age	79.3%	64.8%
Complete vaccination coverage (BCG, PENTA 1–3, polio 1–3, measles) among children 12- < 24 months of age	73.6%	56.6%

*Note*: These data and the methodology for obtaining them are described more fully elsewhere [3]

## Abbreviations

AMTSL: Active management of the third stage of labor
BCC: Behavior change communication
CBIO: Census-Based, Impact-Oriented Approach
CI: Confidence interval
CSRA: *Consejo de Salud Rural Andino*
DHS: Demographic and Health Survey
iCCM: Integrated community case management (of childhood illness)
LMICs: Low- and middle-income countries
LiST: Lives Saved Tool
MMR: Maternal mortality ratio
MSPAS: *Ministerio de Salud Pública y Asistencia Social* (Ministry of Health and Social Assistance)
NNMR: Neonatal mortality rate
PY: Project Year
U5MR: Under-5 mortality rate
UNICEF: United Nations Children’s Fund
WHO: World Health Organization
